# Mining: South Africa's legacy and burden in the context of occupational respiratory diseases

**DOI:** 10.3402/gha.v6i0.20512

**Published:** 2013-02-21

**Authors:** Rajen N. Naidoo

**Affiliations:** Discipline of Occupational and Environmental Health, University of KwaZulu-Natal, Durban, South Africa

Mining has played a central role in the development of South Africa – from the importation of European labour through to the migrant labour system that characterised apartheid, our mineral resources have molded our political, social, and economic paradigms over the last 140 years. The current turmoil on the mines emphasises this legacy and its centrality in our history. The mining industry not only shaped the early development of legislation to protect the health of workers in the 1900s, but it also allowed South African researchers to produce groundbreaking research in mining-related respiratory diseases throughout the last century. Gill Nelson's work is located within this context and provides new directions for mining-related occupational respiratory diseases.

The history of mining in South Africa dating back to the late nineteenth century is synonymous with silica exposure, silicosis, and tuberculosis. Under pressure to protect the large number of the newly arriving European mineworkers, following the conclusions of the 1902 Milner Commission of Enquiry into Phthisis (1), the Miners’ Phthisis Act of 1910 was promulgated (2). Despite this being among the earliest worker health protection legislation globally, the mining industry, over the subsequent 100 years, has contributed to substantial mortality and morbidity of miners. This prompted in recent times, the Leon Commission of Enquiry, revised legislation, including the Mine Health and Safety Act and national and international court challenges – all addressing the concerns of work-acquired respiratory diseases.

Mine related respiratory diseases have been an international concern, with silicosis at the forefront, leading to the Global Programme for the Elimination of Silicosis (3). It is estimated that pneumoconiosis accounts for 30 000 deaths annually, with approximately 1.3 disability-adjusted life years (3). The prevalence rates in South Africa are equally of concern: the studies of ex-miners from Botswana, Transkei, and Lesotho, suggested prevalence rates of pneumoconioses ranging from 26% to 36% of former mineworkers (4–6). Faced with such data, the South African Department of Labour implemented the National Programme for the Elimination of Silicosis (7). As recognised in the latter programme, control of occupational exposures remains the mainstay of addressing the problem. The National Programme intends to eliminate silicosis by 2030 and progressively reduce the current occupational exposure limit to 0.025 mg/m^3^.

In a country ravaged by the HIV/AIDS epidemic, silica exposure assumes far greater importance (8). The mining legacy referred to above creates the very environment in which communicable diseases flourish: poor housing and living environments, overcrowding, lack of access to appropriate health services, etc. Silica-exposed workers who are HIV positive are at multiplicative risk for developing tuberculosis, compared to those without silica exposure (9), and the multidrug resistant forms of the disease. The trends identified by Nelson in her research presents a cause for concern. The almost 10-fold increase, the absolute prevalence rate of 32%, and the two-fold racial difference of silicosis among impoverished miners (10) clearly indicate that their risk for acquiring TB is substantial. If these workers are then returning home to their distant homelands (in and around South Africa), without the appropriate access to health care, the struggle against TB becomes almost insurmountable – thus making the control of silica dust in our mines a broader public health issue, and not just one for the mining industry.

Surveillance for occupational diseases in South Africa is poor. However, the Pathology Automation Service (PATHAUT) remains a significant exception to this. PATHAUT is one of the leading autopsy surveillance systems globally and is a major resource for researchers. PATHAUT has assumed almost legendary status in the world of occupational respiratory disease research, thanks to iconic researchers such as JC Wagner and his fellow workers, who, using the early guises of the autopsy service, described the first ever autopsy cases of mesothelioma (11) and progressive massive fibrosis among coalworkers (12). PATHAUT has allowed us to better understand the relationship between occupational exposures and diseases in the mining industry, particularly in goldmining (13), and to some extent in coal (14). PATHAUT does have some inherent biases, particularly in the way miners present to autopsy. This varies across the different mining entities, and racial groups and residential areas (15). However, these biases are unlikely to have impacted on the findings by Nelson, and her use of this database provides much confidence in the data that she presents.

The interesting findings of asbestos-related diseases among diamond miners and silicosis among platinum miners are worthy of further investigation. The low silica exposures seen in the platinum industry at levels below international silica occupational exposure levels, and concentrations of silica content not exceeding 1% probably accounts for the very low prevalence rates reported by Nelson (adjusted rates ranging from 0.4% to 0.06% depending on the definition). This compares to the low levels of silicosis seen on coalmines, with radiological prevalences ranging from 2 to 4% (16), and autopsy prevalence rates of 10.9%, with reported silica levels of 0.07 mg/m^3^ (14).

Although the numbers of asbestos-related diseases were expectedly low, given the rigorous review of exposure data adopted by Nelson, it is interesting that there were, in actual fact, ANY cases with asbestosis or asbestos pleural disease – both of which require substantial exposure over a long period of time. While cases of mesotheliomas have been documented in environmentally exposed people, asbestosis itself cannot be attributed to environmental exposure. Nelson provides exposure information to support the assertion that this is work-related.

The studies by Nelson build on our depth of existing knowledge of mine related respiratory diseases, particularly in South Africa. Her findings on asbestos related diseases among diamond miners and silicosis among platinum miners, whilst very preliminary, forces researchers to have open minds when investigating known diseases or known exposures. Certainly, the findings of disease in these miner populations are small and the assessment of exposure limited, but clearly the findings suggest the need for more work. Nelson implies that if we do not monitor for these outcomes, we are not likely to detect them. As Nelson points out, in order to ensure these diseases are not missed, we need to improve our medical surveillance programmes, and more importantly, better describe exposure in these working populations. Mining companies have a responsibility of assessing the different types of exposure, and ensuring that exposure levels are monitored and effectively controlled as a first step to addressing our epidemics of silicosis and mine-related respiratory diseases.
